# Fatal Poisoning from Sugar Plums

**Published:** 1853-03

**Authors:** 


*Fatal Poisoning from Sugar Plums.*—At the meeting, Saturday evening 20th November, Professor J. L. Riddell stated that, at the request of Dr. Geo. E. Harral, he had examined a handful of variously painted sugar plums. They were globular, about half an inch in diameter, and with a verrucose surface, somewhat resembling strawberries. About one-fourth of them wore upon the surface a deep orange yellow color, which was found to be due to *chromate of lead.*

The case of poisoning occurred as follows:

At 11, A. M., on the 18th instant, the slave girl, æt. 7, in perfect health, belonging to Mrs. Connellin, 50 Carondelet street, accompanied her mistress to the Poydras Market. A confectioner of that neighborhood, of whom Mrs. C. made some purchase, gave the girl a liberal handful of the sugar plums alluded to, which she ate. In the afternoon the child complained of headache, and had slight attacks of vomiting. Toward evening, the mistress becoming alarmed, sent for Dr. Harral, who responded promptly to the call, but arrived a minute or two after the child had ceased to breathe. Another handful of variegated sugar plums, was, without explanation, procured from the same jar, and submitted for examination, as alluded to.

Professor R. suggested for consideration, whether the Society should not recommend the enactment of a city ordinance, aiming to prevent the sale of poisonous confectionery.

Professor Hunt stated that he had often observed the pernicious effects of poison candy in young children. He had seen distressing symptoms arise from candy colored with gamboge. He thought the attention of the city authorities should be called to the subject.

Dr. Fenner thought it entirely useless for the Society to trouble itself, for he felt assured, from past experience, that the city authorities could not be induced to notice the matter.—*Trans. of the Physico-Medical Society.—New Orleans Monthly Med. Register.*

